# A novel algorithm for detecting multiple covariance and clustering of biological sequences

**DOI:** 10.1038/srep30425

**Published:** 2016-07-25

**Authors:** Wei Shen, Yan Li

**Affiliations:** 1 Medical Research Center, Southwest Hospital, Third Military Medical University, Chongqing, 400038, China; 2 Department of Microbiology, College of Basic Medical Sciences, Third Military Medical University, Chongqing, 400038, China

## Abstract

Single genetic mutations are always followed by a set of compensatory mutations. Thus, multiple changes commonly occur in biological sequences and play crucial roles in maintaining conformational and functional stability. Although many methods are available to detect single mutations or covariant pairs, detecting non-synchronous multiple changes at different sites in sequences remains challenging. Here, we develop a novel algorithm, named Fastcov, to identify multiple correlated changes in biological sequences using an independent pair model followed by a tandem model of site-residue elements based on inter-restriction thinking. Fastcov performed exceptionally well at harvesting co-pairs and detecting multiple covariant patterns. By 10-fold cross-validation using datasets of different scales, the characteristic patterns successfully classified the sequences into target groups with an accuracy of greater than 98%. Moreover, we demonstrated that the multiple covariant patterns represent co-evolutionary modes corresponding to the phylogenetic tree, and provide a new understanding of protein structural stability. In contrast to other methods, Fastcov provides not only a reliable and effective approach to identify covariant pairs but also more powerful functions, including multiple covariance detection and sequence classification, that are most useful for studying the point and compensatory mutations caused by natural selection, drug induction, environmental pressure, etc.

Although basic biological principles, such as the fidelity of replicases and the compatibility of codon degeneracy, attempt to maintain the stability of genes and proteins, random point mutations inevitably provide innumerable novel opportunities for genetic selection and evolution. The accumulation of non-synonymous mutations promotes the formation of homologies, which enable structural diversity while maintaining similar function. Thus, the homogeneity and heterogeneity of proteins hold great interest for the study of structure-based function and molecular evolution.

To this end, many bioinformatics tools have been created. Multiple sequence alignment (MSA) is widely used to identify sequence similarity^1^,^2^, and extended methods such as motif search tools and phylogeny trees have been developed to explore functional regions and evolutionary relationships. Although conserved regions among orthologues and paralogues are considered core structures required for basic biological function^3^, co-variant amino acids have attracted attention recently for their roles in differentiation and phenotype, which reflect a subtle correlation between the polymorphism and stability of proteins^4^,^5^. Several algorithmic approaches have been used to explore the coevolution of protein residues, such as explicit likelihood^6^, maximum likelihood^7^, joint probability estimation^8^, and correlation coefficient tests^9^. However, many of these methods are unable to separate phylogenetic linkages from covariant sites in a group of mixed sequences^9^,^10^ that might feature multiple or dynamic evolutionary issues. Furthermore, several of these methods do not effectively remove noise derived from unrelated sequences and ignore Indel fragments in the alignment. Recent effective methods such as PSIcov and DCA^11^,^12^ detect only covariant pairs and not multiple covariance. In addition, due to inadequate consideration of the relationship between a protein and its ligand, previous estimations of covariance are not reliable depending on adjacent amino acids in a 3D structure. Thus, there is no good standard for testing covariance, and most models neglect computing efficiency.

In this work, we attempt to establish a novel approach to rapidly and effectively detect multiple covariance in a dataset of sequences to interpret the profile of biological divergence and its significance.

## Methods

### Data initialization

Amino acid (or nucleotide) sequences aligned by MSA were used as input data. Initially, this model discarded illegal sequences and unrelated sites according to the following criteria: a) more than one third of residues (or bases) in a sequence matched the consensus sequence; and b) at least two types of residues appeared at one site in at least 5 similar samples.

### Independent pair model

We designed a new model to detect independent covariant pairs based on direct correlation. First, one residue at one site was defined by a site-residue element. For instance, the *x(m)* element represented a residue *m* at site *x*, and the *y(n)* element represented a residue *n* at site *y*. For any two sites, the purity of the coupled elements *x(m)y(n)* was calculated as a correlation probability, the purity value was denoted by *P*, and the association of every pair was defined as:


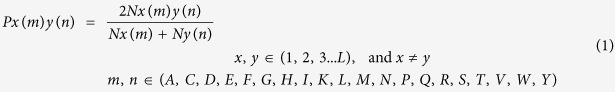


The length of the aligned sequence is denoted by *L*, and the number of elements (or pairs) is denoted by *N*.

As a correlation function, a rational threshold of *P*_*x(m)y(n)*_ should be considered first. According to the formula, if there are no coupled elements, *P*_*x(m)y(n)*_ is zero, and if *x(m)* is completely coupled with *y(n), P*_*x(m)y(n)*_ is one. When all of *x(m)* are coupled with half of *y(n)*, *P*_*x(m)y(n)*_ is 2/3, a correlation can be observed with *P*_*x(m)y(n)*_greater than 2/3. Thus, the rational threshold of purity ranges from 2/3 to 1 (2/3 < P≤ 1), with a larger value representing a stronger correlation. As with real biological events, covariance does not occur at one time point, and the purity of covariance is not always 100%; thus the default threshold value was set to a appropriate correlation value of 0.7 (Figure S1). Moreover, because covariance results in a stable transformation of amino acids, at least two types of elements are needed to confirm covariance between the two sites.

### Correlated tandem model

**Figure 1:**
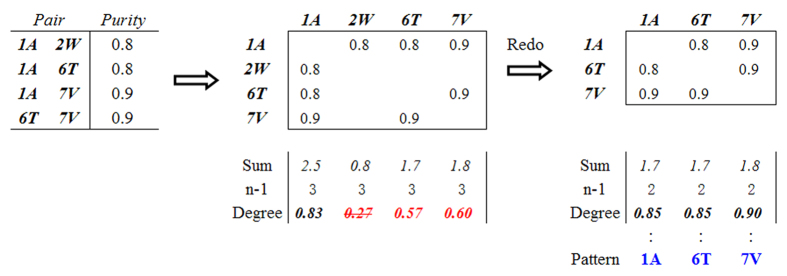
Schematic of the correlated tandem model.

To detect multiple covariance information, we joined the independent pairs with each other in a potential tandem pattern if they exhibited a common site-residue element. First, the association degree of each element was evaluated to confirm a reliable pattern. Figure 1 demonstrates the processing procedure.

Based on the correlation of the inter-restriction among the elements, the associated independent pairs were harvested into a group and transformed into a matrix. The average purity of each column or row reflects the degree of the general association of the element compared to others. Here, the number of columns or rows in the current matrix is denoted by *n*, the purity value of every pair is denoted by *P*, the degree of association of each element is individually calculated, and the smallest association is obtained by the following formula:


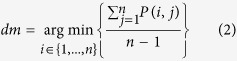


If *dm* is lower than a threshold, the corresponding element is removed from the current matrix. Then, the model re-calculates the matrix until the degree value of every element is higher than the threshold.

For example, if the threshold of the degree of association is set to a intermediate correlation degree of 0.7, the ‘2 W’ element with a *d*_*m*_ (the smallest association) less than 0.7 is removed firstly during the first round of calculation, then the degrees of every elements sites are more than 0.7 during the next round. So we successfully harvest a covariant pattern of ‘ATV’ at 1, 6 and 7, which is represented in a (*1-A 6-T 7-V*) format. This model then detected other patterns in subsequent groups in an identical manner. Finally, the confirmed patterns are reported after removing the subsets of all patterns.

### Sequence clustering

Based on the covariant patterns, we can easily cluster the sequences. Because every pattern represents a strong characteristic of one population, a loose fitness of the pattern is sufficient to classify a sequence into the relevant population. Using the distribution test of pattern matching proportion, a sequence was classified if 45% or more of the pattern sites matched (Figure S2). This process can be used for genotyping and evolutionary analysis, among other applications.

### Algorithm implementation

These models were integrated into a new algorithm named ‘Fastcov’ implemented in the golang ( https://golang.org) programming language. The executable binary files for most popular operating systems are freely available at http://yanlilab.github.io/fastcov.

### Testing and verification

Well-established data, such as reverse transcriptase (RT) data for hepatitis B virus (HBV), glyceraldehyde 3-phosphate dehydrogenase (GAPDH), the 90 Kd heat shock protein (Hsp90) and toll-like receptor 4 (TLR4), were adopted as samples for model testing and performance verification. All sequences were downloaded from public biological databases. The MSA data were prepared using the ClustalW tool, and a phylogenetic tree was drawn using the maximum likelihood method with default parameters in Mega 7.0 software^13^. The results obtained using our new algorithm were evaluated based on the structure of the phylogenetic tree and the genotyping performance, based on the background information provided with the samples. Several evaluation indexes, Sensitivity (Sen), Specificity (Spe), and Accuracy (Acc), were used to evaluate the performance^14^.

Moreover, ten-fold cross validation was used to estimate the model’s ability to fit out-of-sample data. During the procedure, the dataset of each genotype was randomly divided into 10 subsets marked with No. 1 ~ 10. The subsets with same number from all genotypes were put together as a test data, and the remaining sets composed a corresponding training set. After the tests were performed on the 10 groups of training-testing datasets, the average indexes across all the trials were computed. Furthermore, the dataset was randomly sampled in a proportion of 50% or 10% for different scale test.

## Results

### Genotyping performance based on covariant patterns

Eight genotypes (A–H) of Hepatitis B virus (HBV) have been identified worldwide and exhibit distinct geographical distributions. The gold standard for classifying any virus depends on the sequence divergence of the entire genome. HBV has been classified into genotypes A-D based on the intergroup divergence of the entire genome, which exceeds 8%^15^,^16^.

Here, we assessed the performance of Fastcov by focusing on one HBV protein. For reliable detection, non-redundant data were obtained from a professional HBV database^17^. In total, 3424 amino acid sequences of the HBV polymerase RT region from genotypes A-D were prepared. To avoid bias due to inadequate sampling, genotypes E-H were not considered in this study. Using our Fastcov algorithm with a purity threshold of 0.7, five groups of tandem sites with 21 patterns were obtained (Table 1). Among them, 4 covariant patterns exhibited typical genotype characteristics by which the relevant sequences could be clustered into their own genotype group (Table 1, bold font). The number of sequences labelled by genotyping was counted in every cluster to evaluate performance. The accuracies reached 98% and above. In Table 1, the evaluation indexes indicate a satisfactory clustering effect for every genotype, indicating that the covariant patterns for the RT protein were consistent with genotyping. Our data also suggest that divergence in the genome contributed to consistent changes and adaptive evolution at the protein level. This result further supports that structural and functional requirements determine selection, as previously reported^18^. The other two patterns observed at the tandem sites 124-145-151-322 separated genotype AB from genotype CD (Table 1, bold font), but no patterns separating genotypes AC and BD were observed.

In addition, to confirm the reliability of Fastcov, we performed 10-fold cross-validation on three scales of HBV sequences. According to the best patterns observed for types A, B, C and D, the multi-class classification results were represented by the average value of the four genotypes^14^. These results demonstrated that the performance of the Fastcov algorithm was highly reliable (Table 2). We therefore suggest that the Fastcov algorithm not only possesses outstanding power for covariant analysis but is also reliable for evolutionary classification based on covariant patterns.

### Comparison of the performance of different algorithms

To evaluate the performance of Fastcov, we performed a comparative analysis with the most popular relevant methods: PSIcov and CAPS^11^,^19^. The analysis was performed in a Windows 7 64-bit operating system with an Intel i5 4570 3.2 GHz 4-core CPU and 8 G memory using the above dataset of 3424 amino acid sequences with a length of 344 residues. The key indexes, including computational efficiency, and their functions were evaluated. The test results, shown in Table 3, indicate that Fastcov is very fast, occupies less memory, and exhibits more powerful functions than the other methods.

**Figure 2:**
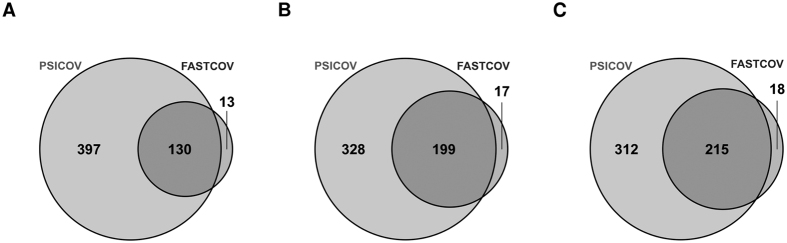
Venn diagrams of co-pairs produced by Fastcov and PSIcov. (**A**) At a threshold of 0.7 in Fastcov. (**B**) At a threshold of 0.5, with purity of one pair more than 0.8 in Fastcov. (**C**) Union of A and B in Fastcov.

As shown in Table 3, because the latter two tools were unable to perform multiple covariance detection or sequence classification, they were incompatible with all but the co-pair function of Fastcov. We therefore examined the intersection of the co-pairs produced by Fastcov and PSIcov. The Venn diagram indicated that Fastcov and PSIcov obtained similar results at the level of co-pair detection (Fig. 2A). If the threshold decreased to 0.5 below the bottom line of the rational range, and those sites where one pair with P ≥ 0.8 remained, the intersection between the Fastcov and PSIcov datasets increased (Fig. 2B), indicating that the models were similar. According to the suggested parameters, PSIcov listed too many candidate pairs following manual reselection^11^.

It should be noticed that the information of pairs provided by PSIcov is incomplete, which only contains sites but no residues, so that people don’t know what covariance occurred in the sites. By contrast, the Fastcov presents complete information in form of the site-residue elements. Because a covariance event between the two sites would lead to at least one transformation of a pair of amino acids into another pair, that means there should exist at least two types of amino acids pairs, so Fastcov confirms a covariance when at least two types of elements reached the threshold simultaneously. If only one, transformation could not take place, then these pairs were considered noise to discard by Fastcov. Unfortunately, they were retained by PSIcov, exactly locating in the subsection of PSIcov in Fig. 2.

On the other hand, after checking the Fastcov subsection, we found that the covariant pairs with lower frequency were ignored by PSIcov. It suggested that FASTcov is better than PSIcov to deal with low frequency data.

Moreover, despite a result similar to that of CAPS at the co-variance level, the co-evolutionary amino acid group reported by CAPS could not distinguish the genotypes. This result indicates that CAPS was unable to provide a precise evolutionary classification.

Thus, only Fastcov retained the sequence and amino acid information in the result, which facilitates the interpretation of the data by biologists. Unfortunately, some algorithms, such as DCA, did not provide software for comparison^12^.

Furthermore, the versions of Fastcov for popular operating systems, including Windows, Linux, Mac OS X, and BSD, are adequate. Fastcov is easy to use and install; it must simply be downloaded and run in a command line terminal. With the exception of the optional parameters, only one input file containing the MSA sequences in FASTA format is needed to run the analysis.

### Covariant features of GAPDHs corresponding to the phylogenetic tree

GAPDH is a classic glycolytic enzyme that plays fundamental roles in energy metabolism and non-metabolic processes, including transcription activation and the initiation of apoptosis. GAPDH is related to a variety of pathologies, including diabetes and age-related neurodegenerative disorders^20^,^21^,^22^. GAPDH is also a well-known house-keeping gene that is widely used as an internal control in studies involving protein quantification due to its highly conserved structure and stable expression in tissues^23^.

**Figure 3:**
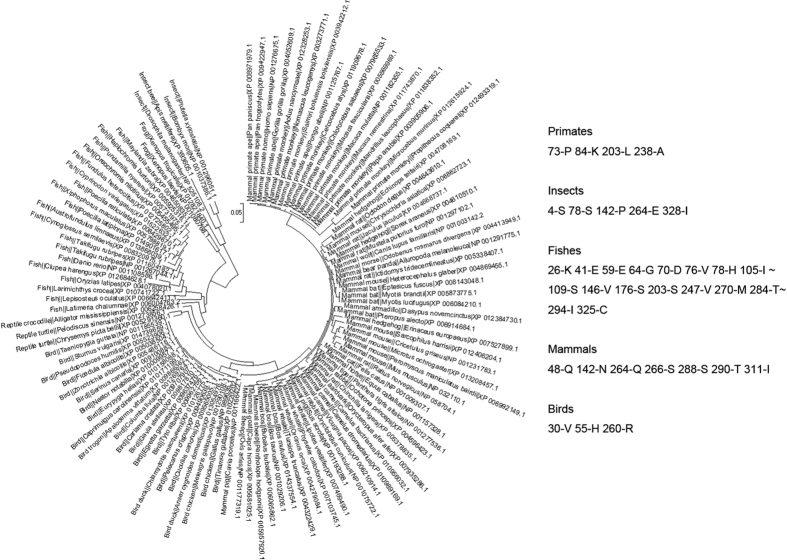
Covariant patterns corresponding to different families in the phylogenetic tree. Note: The sites are denoted by the order of the aligned data.

Here, we characterized the diversity among GAPDHs in different animal families. In total, non-redundant amino acid sequences from 112 species were retrieved from the Refseq database. Based on these sequences, Fastcov successfully detected family-characteristic covariant patterns that corresponded perfectly to relevant branches in the phylogenetic tree (Fig. 3), thus providing new insights for the elucidation of gene evolution and structural diversity.

### Covariant features of HSPs corresponding to the phylogenetic tree

**Figure 4:**
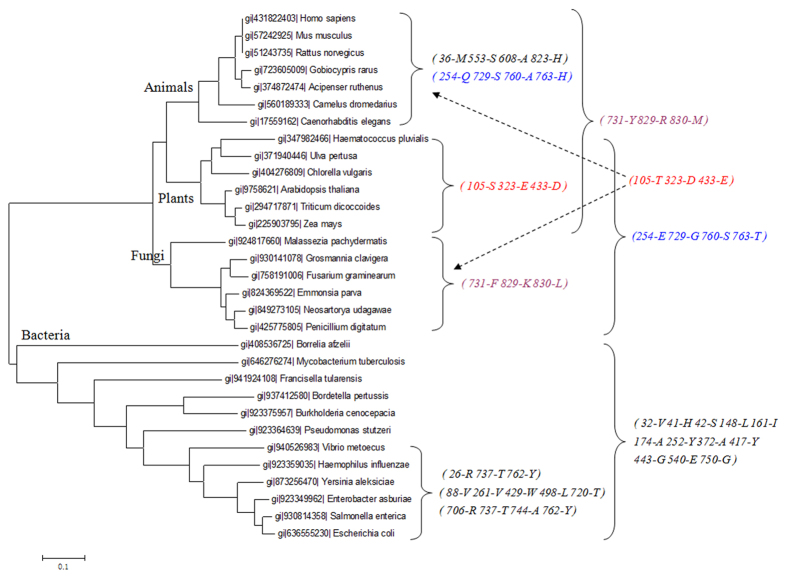
Covariant patterns corresponding to different families in the phylogenetic tree. Note: The sites are denoted by the order of the aligned data.

Heat-shock proteins (HSPs) are stress proteins that are highly conserved and present in virtually all living organisms. HSP90 proteins are ancient and are conserved from bacteria to mammals^24^,^25^,^26^. To observe covariant patterns during evolutionary processes, 31 amino acid sequences from bacteria, fungi, plants and animals were prepared for examination. Our data revealed that HSP90 was relatively conserved, with many Indels in sequences from different organisms. Fastcov was therefore run under a purity threshold of 0.95, and sequences with genotype characteristics are shown in Fig. 4. We detected more frequent and longer patterns in bacterial groups, which displayed covariances occurring with a higher frequency. By contrast, in eukaryotes, covariances always occurred in relatively conserved tandem sites. For example, the pattern (254-Q 729-S 760-A 763-H) distinguished animal sequences from plants and fungi, and the pattern (105-S 323-E 433-D) differentiated plants from animals and fungi. Fastcov indicated that the multiple-site combined variance was a common mode utilized during evolution and thus provides a new method by which to assess co-evolutionary modes.

### Divergence analysis of TLR4

Toll-like receptors (TLRs) are highly conserved from *Drosophila* to humans and share structural and functional similarities that play fundamental roles in pathogen recognition and the activation of innate immunity^27^. Human and mouse TLR4s both recognize LPS but occasionally exhibit differential activation^28^. Selective recognition of temperature-dependent shifts in LPS acylation by human and mouse TLR4 was recently reported^29^. To clarify the molecular mechanism underlying this phenomenon, we performed a divergence analysis of TLR4 using the Fastcov approach.

**Figure 5:**
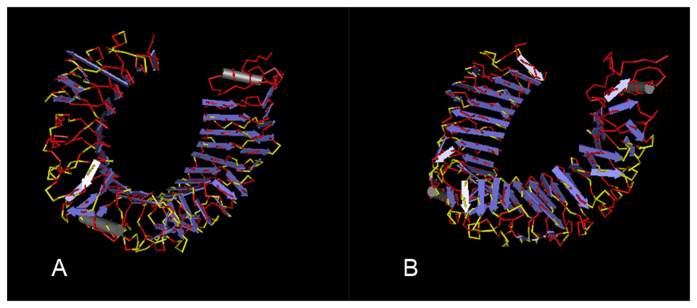
Distribution of covariant sites (yellow) between human and mouse TLR4 (**A**) left view; (**B**) right view.

As reported by the RCSB Protein Data Bank (PDB), the three-dimensional (3D) structures of human and mouse TLR4 include a similar C-shaped circle. Interestingly, among the 570 amino acids, nearly two thirds were conserved (red in Fig. 5); Fastcov exposed a covariant pattern of 180 sites, of which more than 90% (yellow) were located in the loops at the outside of the circle, with few in the beta sheets at the inner region. This result suggests that the divergence is mainly associated with the stability of the 3D structure, providing a clue to understand the differential activation of TLR4 from different species at the molecular level^28^,^29^.

## Discussion

Covariant mutations are crucial for maintaining the structural characteristics of a protein and, consequently, conformational and functional stability^30^. Accumulating data from numerous populations has provided expanded insights on the significance of multi-site combined mutations.

Point mutations are typically stochastic, and as mutations accumulate, a covariant pattern appears in the population. With multiple coincidences, biological trends become visible. During the process, mutations only provide diversity, but selection shapes the pattern. In this study, we developed a novel algorithm to identify correlated changes using an independent pair and correlated tandem model. This algorithm is based on the idea of correlated inter-restriction, which is suitable for the analysis of non-synchronous co-variances. This algorithm enables the reliable detection of multiple-residue covariant patterns and sequence clustering, which provides a new method for studying the divergence of biological sequences.

Substitutions or conservations at two independent sites are not considered directly comparable due to differences in their amino acid compositions^9^. To resolve this issue, Fastcov utilizes a new independent pair model that considers site-residue elements. Fastcov provides faster processing as well as more powerful functions than other complex methods. The lightweight Fastcov algorithm significantly improves processing efficiency and thus could easily be applied to much larger datasets. Furthermore, the Fastcov algorithm can detect multiple covariances and perform sequence classification, in contrast to existing algorithms.

Sample size is a common factor influencing signal and statistics. Fastcov is adequate for datasets with samples of different sizes, as shown in Section 1 of the results. Furthermore, Fastcov opens the parameters to accommodate observation zooms, higher threshold presents patterns with lower heterogeneity, and lower one would provide more patterns including those with higher heterogeneity. In particular, Fastcov adopts several strategies to eliminate noise, including inter-restriction model design, limitation to the smallest sample size, and fast processing of large datasets. For any method of covariance analysis, well-aligned data are necessary for a precise result.

CAPS is designed to remove phylogenetic co-evolution, which effectively distinguishes background correlation from true correlation^9^, in contrast to many other algorithms^31^,^32^. For various reasons, Fastcov focuses on detecting co-variance by site-residue elements. However, Fastcov is able to obtain more detailed covariant information by relying on reference sequences. In contrast to interaction methods^33^, the Fastcov approach is not typically used to identify protein-protein interaction interfaces and is unable to resolve the docking problem. However, the information reported by Fastcov might be useful for extended studies when accompanied by relevant tools.

Fastcov is most useful for studying the point and compensatory mutations caused by natural selection, drug induction, and environmental or host immunological pressure and is less useful for analysing the fragments of genes with frequent recombination, such as hemagglutinin and neuraminidase genes of the influenza A virus.

Finally, the samples used in this study were chosen primarily to assess the performance and applications of this model. Additional biological information could be explored by Fastcov by employing a comparative approach under various conditions according to the needs of the researcher.

In conclusion, Fastcov provides a fast and reliable approach for multiple covariance detection and sequence clustering and should be widely used in studies involving sequence diversity analysis, evolutionary classification, genotyping, and protein structure formation. Furthermore, the Fastcov algorithm could be easily implanted into integrated bioinformatics tools as an extended module.

## Additional Information

**How to cite this article**: Shen, W. and Li, Y. A novel algorithm for detecting multiple covariance and clustering of biological sequences. *Sci. Rep.* **6**, 30425; doi: 10.1038/srep30425 (2016).

## Supplementary Material

Supplementary Information

## Figures and Tables

**Table 1:** Clustering performance by covariant patterns.

Cluster	Seq_num	Genotype	Accuracy	Matched genotypes	Patterns
3	594	A	*98.99%*	A: 588, B: 0, C: 0, D: 0	*53-I 103-I 139-Q*
1	594	A	*93.81%*	A: 387, B: 2, C: 1, D: 2	*163-V 217-R 253-I*
2	594	A	*91.61%*	A: 483, B: 0, C: 0, D: 2	*7-D 103-I 122-N 126-Y 139-Q 153-W 163-V 217-R*
20	2830	BCD	*98.89%*	A: 34, B: 961, C:1116, D: 749	*7-T 103-V 122-I 126-H 139-N 153-R 163-I 217-L 253-V*
21	2830	BCD	*97.43%*	A: 84, B: 961, C:1116, D: 749	*122-I 126-H 139-N 153-R 163-I 217-L 253-V*
8	961	B	*99.69%*	A: 0, B: 958, C: 0, D: 0	*13-R 16-T 127-R 222-A 238-H*
16	2463	ACD	*99.84%*	A: 594, B: 0, C:1112, D: 753	*13-H 16-I 127-G 222-T 238-N*
9	1116	C	*99.37%*	A: 0, B: 1, C:1104, D: 0	*53-S 55-H 109-P 118-T 121-N 131-D 223-S 224-I*
7	1116	C	*83.06%*	A: 0, B: 0, C: 927, D: 0	*13-N 223-S 224-I*
15	2308	ABD	*99.53%*	A: 594, B: 960, C: 15, D: 753	*53-N 55-R 109-S 118-N 121-I 131-N 223-A 224-V*
6	753	D	*99.47%*	A: 0, B: 0, C: 0, D: 749	*7-A 122-F 149-Q 257-Y 259-S 266-I*
4	753	D	*98.41%*	A: 0, B: 0, C: 0, D: 741	*54-Y 122-F 149-Q 257-Y 259-S*
5	753	D	*99.31%*	A: 2, B: 0, C: 1, D: 739	*256-C 257-Y 259-S*
19	2671	ABC	*99.18%*	A: 579, B: 961, C:1111, D: 8	*54-T 122-I 149-K 248-N 257-W 259-T*
18	2671	ABC	*98.10%*	A: 594, B: 961, C:1070, D: 19	*149-K 257-W 259-T 266-V*
17	2671	ABC	*98.10%*	A: 589, B: 955, C:1069, D: 7	*256-S 257-W 259-T*
10	1555	AB	*99.53%*	A: 592, B: 953, C: 2, D: 4	*124-N 145-M 151-Y 332-S*
11	1555	AB	*99.33%*	A: 593, B: 959, C: 18, D: 2	*145-M 151-Y 221-Y 317-A 332-S*
12	1555	AB	*98.89%*	A: 593, B: 961, C: 21, D: 16	*16-T 145-M 151-Y 221-Y*
14	1869	CD	*99.79%*	A: 0, B: 1, C:1112, D: 752	*124-Y 145-L 151-F 332-C*
13	1869	CD	*98.94%*	A: 0, B: 1, C:1089, D: 751	*145-L 151-F 221-F 317-S 332-C*

*Note: the sites are denoted by the order of the aligned data.*

**Table 2:** Ten-fold cross-validation of performance for datasets of different scales

Data Size	Seq num	Avg.sensitivity	Avg.specitity	Avg.accurracy
*100%*	*3,424*	*99.24* ± *0.34%*	*99.99* ± *0.03%*	*99.80* ± *0.10%*
*50%*	*1,714*	*99.12* ± *0.60%*	*99.98* ± *0.05%*	*99.77* ± *0.15%*
*10%*	*344*	*98.02* ± *2.95%*	*99.20* ± *0.26%*	*99.53* ± *0.72%*

**Table 3:** Performance of different algorithms

	*Speed*	*Memory occupation*	*Multiple threads*	*Co-pair detection*	*Multiple covariance detection*	*Sequence classification*	*Sequence and amino acid information*
Fastcov	*14* *s*	*1%*	*Yes*	*Yes*	*Yes*	*Yes*	*Remaining*
PSIcov	*2* *m35s*	*9%*	*Yes*	*Yes*	*No*	*No*	*Lost*
CAPS	*≫ 40* *m*	*Out of memory*	*No*	*Yes*	*Yes*	*No*	*Lost*
