# Supplementary material for: A novel algorithm for detecting multiple covariance and clustering of biological sequences
Source: Sci Rep. 2016 Jul 25;6:30425. doi: 10.1038/srep30425 (PMC4958985; doi:10.1038/srep30425)
Supplement: Supplementary Information [file srep30425-s1.pdf]

Supplementary Data

A novel algorithm for detecting multiple covariance and clustering of biological sequences

Wei Shen [1,2], Yan Li [1,2,*]

1. Medical Research Center, Southwest Hospital, Third Military Medical University, Chongqing, 400038, China.
2. Department of Microbiology, College of Basic Medical Sciences, Third Military Medical University, Chongqing, 400038, China.

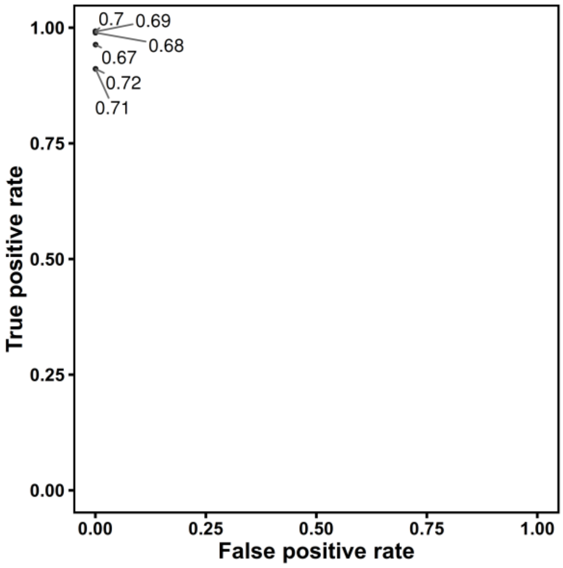

Figure S1. The estimation of the parameter sensitivity

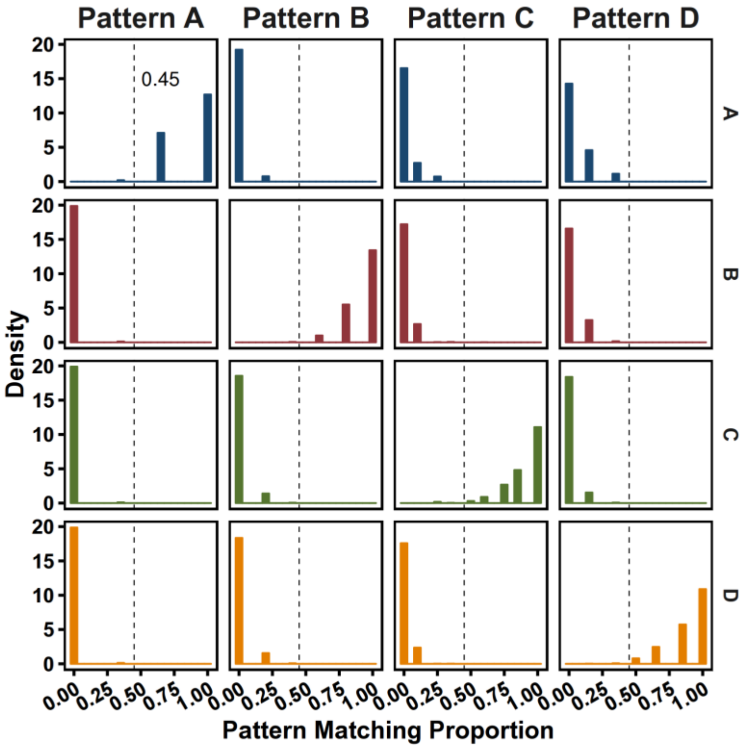

Figure S2. The pattern matching proportion distribution. Pattern matching proportion (PMP for short) is defined as the proportion of matched site-residue elements from one sequence on one pattern. Dashed lines indicate the PMP threshold used for classification. For one sequence, a pattern is added to candidates of predicting groups if the PMP exceeds the threshold. The predicting group is then assigned to the group with the maximum PMP.
